# Intermediate- and Long-Term Exposure to PM_2.5_ and Its Chemical Components in Relation to Nocturnal Sleep Duration and Daytime Napping Duration

**DOI:** 10.3390/toxics14050437

**Published:** 2026-05-14

**Authors:** Lidan Hu, Xiuhua Yan, Xinhui Qiu, Zhiyuan Li

**Affiliations:** 1 School of Public Health (Shenzhen), Sun Yat-sen University, Guangzhou 510275, China; 2 Intelligent Sensing and Proactive Health Research Center, Sun Yat-sen University, Shenzhen 518107, China; 3 Shenzhen Key Laboratory of Pathogenic Microbes and Biosafety, School of Public Health (Shenzhen), Shenzhen Campus of Sun Yat-sen University, Shenzhen 518107, China

**Keywords:** sleep duration, linear mixed-effects models, Qgcomp, restricted cubic splines, China

## Abstract

While the association between criteria air pollutants and sleep duration is well-documented, evidence on the impact of fine particulate matter (PM_2.5_) chemical components on sleep remains limited. This study investigated the effects of intermediate- (6-month) and long-term (2-year) exposure to PM_2.5_ and its five major components—black carbon (BC), organic matter (OM), sulfate (SO_4_^2−^), nitrate (NO_3_^−^), and ammonium (NH_4_^+^)—on nocturnal sleep and daytime napping duration. We included 19,505 participants aged ≥ 45 years from the China Health and Retirement Longitudinal Study (CHARLS, 2011–2018). Residential PM_2.5_ and component concentrations were estimated via the Tracking Air Pollution in China dataset, and sleep data were collected through self-reported questionnaires. Linear mixed-effects models and quantile-based g-computation (qgcomp) were used to assess single- and multi-pollutant effects. Results showed that both intermediate- and long-term exposure to PM_2.5_ components was associated with shorter nocturnal sleep and longer daytime napping. Subgroup analyses revealed greater susceptibility among rural residents, solid fuel users, and individuals without pensions. These findings emphasize the need for component-specific PM_2.5_ control strategies and targeted public health interventions to reduce sleep-related health inequalities, especially in socioeconomically disadvantaged populations.

## 1. Introduction

Good sleep quality is essential, as sleep accounts for approximately one-third of human life [1] and is closely linked to the proper functioning of the brain and bodily systems. Previous studies have focused on sleep issues such as poor sleep quality, abnormal nocturnal sleep duration, insomnia, and sleep onset latency, and have indicated that these problems may increase the risk of cardiovascular diseases [2,3], diabetes [4,5], hypertension [6,7], cancer [8,9], mental health disorders [10,11], and other health outcomes [2,12,13,14,15]. While brief daytime napping can compensate for insufficient nighttime sleep and alleviate daytime sleepiness [16,17], accumulating evidence indicates that excessive daytime napping—often regarded as a marker of underlying sleep disturbances—may correlate with multiple adverse health outcomes, including fatty liver disease [18], dementia [19], stroke [20], and cardiometabolic disorders [21]. Sleep disturbances have emerged as a major public health concern globally, especially in China, where approximately 176 million people suffered from various sleep disturbances in 2019 [22]. Given that women and older adults are more susceptible to sleep disturbances [23,24], the rapid population aging has further exacerbated the substantial disease burden of sleep disturbance across China [22].

Air pollution substantially threatens both physical and mental health [25,26,27]. A growing body of evidence indicates that ambient air pollutants, especially fine particulate matter (PM_2.5_), are linked to adverse sleep disturbances [28,29,30,31,32]. Recent epidemiological studies have highlighted the need to quantify health risks associated with co-occurring air pollutants, including chemical components of particles [25,33]. However, few existing studies [34,35] have specifically evaluated the associations between PM_2.5_ components exposure and sleep disturbances.

While a growing body of evidence links air pollution to adverse sleep outcomes, several research gaps still exist. Firstly, previous studies have mainly focused on the association between criteria air pollutants and adverse sleep outcomes, while no research to date has explored the relationships between PM_2.5_ components and sleep duration, covering both nocturnal sleep and daytime napping. Compared with PM_2.5_ mass concentration, certain chemical components may exert distinct toxicological properties [31,36,37] and show more direct associations with adverse health outcomes [38]. Secondly, most existing studies have neglected potential adverse impacts of PM_2.5_ chemical components on daytime napping duration, a critical indicator of sleep status [39]. Additionally, the relationships between joint exposure to PM_2.5_ components and both nocturnal sleep and daytime napping duration remain largely understudied. Furthermore, findings regarding the association between PM_2.5_ and sleep disturbances remain inconsistent, attributable to differences in data sources, study populations, and data quality [29,31,34,40]. Lastly, most previous studies only adopted a single exposure time window, with few investigating both intermediate- and long-term effects of air pollutants on sleep disturbances.

Consequently, this study aims to examine the associations of intermediate- and long-term exposure to PM_2.5_ components with both nocturnal sleep and daytime napping duration. In addition to single-pollutant models, multi-pollutant models across different exposure windows were applied to assess the effects of PM_2.5_ components’ mixture on sleep duration.

## 2. Methods

### 2.1. Study Population

Data for the present study were obtained from the China Health and Retirement Longitudinal Study (CHARLS), a longitudinal cohort of middle-aged and older adults nationwide in China. CHARLS enrolled participants from 450 communities across 126 cities and counties nationwide. Detailed information on CHARLS is available at the official website (https://charls.charlsdata.com, accessed 6 March 2025). We utilized data from four CHARLS waves, including the baseline survey in 2011–2012 and three follow-up surveys conducted in 2013, 2015 and 2018. A total of 72,201 initial records were retrieved. We then excluded participants with incomplete demographic and behavioral information, individuals aged under 45 years, and those missing sleep duration data. Ultimately, 19,505 participants with 35,832 valid observations were included in the final analytical sample (Figure S1). Ethical clearance for this investigation was granted by the Biomedical Ethics Review Committee of Peking University, Beijing, China (IRB00001052–11015). All participants provided written informed consent prior to enrollment.

### 2.2. Definition of Sleep Duration

Participants reported their sleep habits by answering: “During the past month, how many hours of actual sleep did you get at night?” regarding nighttime sleep, and “During the past month, how long did you take a nap after lunch?” concerning daytime napping. These responses were utilized to quantify two distinct parameters: nocturnal and daytime sleep duration. We eliminated samples with daytime napping duration over 5 h. Individuals reporting exceptionally short (<5 h) or long (>13 h) total sleep time, the sum of nocturnal sleep and daytime napping duration, were also excluded because the reports may be unreliable [41,42].

### 2.3. Exposure Assessment

The concentrations of PM_2.5_ components were acquired from the Tracking Air Pollution in China database (http://tapdata.org.cn, accessed 6 March 2025) at 10 km spatial resolution, which utilized community multiscale air quality simulations, ground measurements, and an extreme gradient boosting algorithm to conduct estimations [27,43]. Monthly concentrations of PM_2.5_ and its components were assigned to participants based on the geographic coordinates of their residential cities. We defined intermediate-term exposure as the 6 mth mean concentration and long-term exposure as the 2 yr mean concentration before each survey. The 6 mth time window was adopted from existing relevant studies [44,45], and its rationality was verified through our preliminary data analysis. Fixed exposure windows were used to calculate 6 mth and 2 yr mean concentrations of black carbon (BC), organic matter (OM), sulfate (SO_4_^2−^), nitrate (NO_3_^−^), and ammonium (NH_4_^+^).

### 2.4. Covariates

Following previous studies [32,46,47], the included covariates are as follows: (1) demographic characteristics, including age, gender, marital status, residence, education level, and pension; (2) lifestyle characteristics, including smoking, drinking, disability, chronic diseases, social activities, cooking, and heating; and (3) meteorological variables, including temperature, relative humidity, and wind speed, which are collected from the National Earth System Science Data Center of China (http://www.geodata.cn, accessed 6 March 2025). Through matching the latitude and longitude of participants’ residential cities, we derived 6 mth and 2 yr mean values of annual mean temperature, monthly mean wind speed, and monthly mean relative humidity preceding the interview date. Table S1 lists the details of all covariates.

### 2.5. Statistical Analysis

Statistical descriptive included median values with interquartile ranges (IQRs) for numerical variables, while qualitative variables were expressed as counts and percentages. Pearson’s correlation was utilized to assess relationships between PM_2.5_ and its components. In addition, restricted cubic splines (RCSs) were applied to obtain the concentration–response relationship between PM_2.5_ components with nocturnal sleep and daytime napping duration.

In single-pollutant analysis, linear mixed-effects models with random intercepts for individual ID and survey waves [29,48] were applied to examine associations between PM_2.5_ components and both nocturnal sleep and daytime napping duration. Given the non-linear relationships between PM_2.5_ components and daytime napping duration detected in RCS analyses, pollutant concentrations were categorized into quartiles for subsequent linear mixed-effects model fitting. For nocturnal sleep duration, model coefficients (β) and corresponding 95% confidence intervals (CIs) were estimated per one IQR increment in PM_2.5_ and its components. For daytime napping duration, pollutant concentrations were modeled across quartiles with Quartile 1 set as the reference group. β and 95% CIs were reported for Quartiles 2 to 4. Stratified analyses were further performed by introducing interaction terms into the linear mixed-effects models, stratified by nine factors: age, gender, smoking, residence, cooking, heating, chronic diseases, pension, and social activities.

Three models were constructed: (1) Model I (crude model) assessed the unadjusted association between each air pollutant and sleep duration; (2) Model II further adjusted for demographic characteristics including gender, age, residence, marital status, smoking, drinking, education, and pension based on the crude model; and (3) Model III additionally incorporated disability, heating, cooking, chronic diseases, social activities, and three meteorological variables to refit the analysis. Model III was designated as the main model.

In multi-pollutant analysis, the “qgcomp” R package was applied to conduct quantile-based g-computation (qgcomp), with adjustment for all covariates, to estimate the combined effect of PM_2.5_ components on sleep duration. This method allows clear interpretation of the relative contribution of each pollutant to the overall association with sleep duration. The coefficient derived from qgcomp represents the change in sleep duration corresponding to a one-quartile increase in mixture exposure.

To verify the robustness of our main results, several sensitivity analyses were performed: (1) defining 1 mth and 3 mth windows as alternative intermediate-term exposure periods, and 1 yr and 3 yr windows as alternative long-term exposure periods; (2) excluding participants who completed only one CHARLS survey wave; (3) excluding smokers, given the well-documented association between smoking and sleep disturbances [49]; (4) for nocturnal sleep duration, reclassifying pollutant concentrations into quartiles with the lowest quartile serving as the reference group.

The computational framework employed R version 4.4.1 software (R Foundation for Statistical Computing, Vienna, Austria) operating on the Windows platform. Multiple imputation was conducted for missing covariates using the “mice” package in R. Two-tailed *p* values < 0.05 were determined as statistically significant.

## 3. Results

### 3.1. Descriptive Statistics

A total of 19,505 participants with 35,832 responses were included, and the median nocturnal sleep duration and daytime napping duration were 7.0 and 0.5 h, respectively. In these 35,832 responses, 20,143 (56.2%) were women, 10,515 (29.3%) were over 65 years of age, and 21,496 (60.0%) were from rural areas. Moreover, 7279 (21.5%) were smokers, 8974 (25.0%) reported drinking more than once per month, 22,146 (61.8%) had one or more chronic diseases, and 5436 (15.2%) received a pension. More details about other basic characteristics are presented in Table 1. Table S2 shows statistics on the intermediate- and long-term mean concentrations of PM_2.5_ and its chemical components and meteorological variables. Additionally, correlations between these PM_2.5_ components are illustrated in Tables S3 and S4.

### 3.2. Nocturnal Sleep Duration Associated with Intermediate- and Long-Term Exposure to PM_2.5_ and Its Components

**Figure 1:**
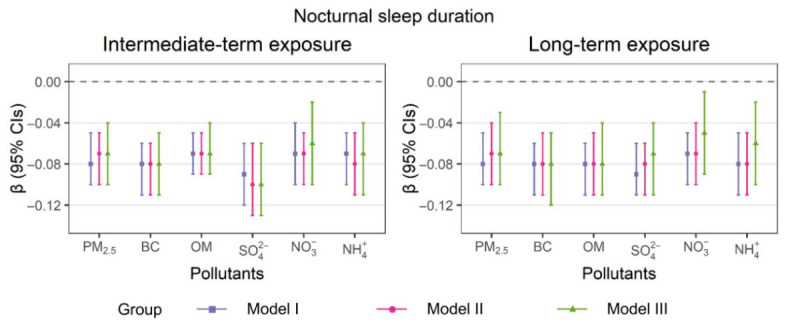
Intermediate- and long-term effects of PM_2.5_ components on nocturnal sleep duration in all models (per IQR increment).

Figure 1 illustrates the associations between individual PM_2.5_ components and nocturnal sleep duration under both intermediate- and long-term exposure scenarios. A consistent pattern of association between PM_2.5_ components and nocturnal sleep duration was observed across the two exposure windows. Across both exposure windows, BC and SO_4_^2−^ showed the most consistent and pronounced negative associations with nocturnal sleep duration. Specifically, the results of fully adjusted models demonstrated that per IQR rise in 6 mth mean concentrations of PM_2.5_, BC, OM, SO_4_^2−^, NO_3_^−^, and NH_4_^+^ decreased nocturnal sleep duration by 0.07 (95% CI: 0.04, 0.10), 0.08 (95% CI: 0.05, 0.11), 0.07 (95% CI: 0.04, 0.09), 0.10 (95% CI: 0.06, 0.13), 0.06 (95% CI: 0.02, 0.10), and 0.07 (95% CI: 0.04, 0.11) h, respectively. A univariate linear mixed-effects model was conducted for each variable in all three models (Table S5).

### 3.3. Daytime Napping Duration Associated with Intermediate- and Long-Term Exposure to PM_2.5_ and Its Components

**Figure 2:**
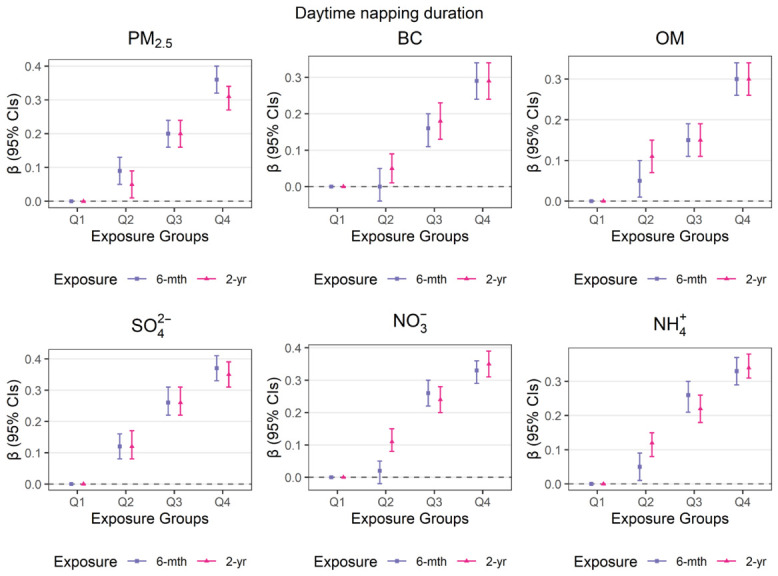
Intermediate- and long-term effects of PM_2.5_ components on daytime napping duration across quartile levels based on Model III.

The association between individual PM_2.5_ component quartiles and daytime napping duration across both intermediate- and long-term exposure windows is displayed in Figure 2. After adjusting for demographic, lifestyle, and meteorological variables, exposure to the highest quartile (Q4) of each PM_2.5_ component was associated with an increase in daytime napping duration compared to the lowest quartile (Q1). Specifically, long-term exposure to the Q4 of PM_2.5_, BC, OM, SO_4_^2−^, NO_3_^−^, and NH_4_^+^ was linked to increases in daytime napping duration, with effect estimates of 0.31 (95% CI: 0.27, 0.34), 0.29 (95% CI: 0.24, 0.34), 0.30 (95% CI: 0.26, 0.34), 0.35 (95% CI: 0.31, 0.39), 0.35 (95% CI: 0.31, 0.39), and 0.34 (95% CI: 0.31, 0.38) h, respectively. Additional details regarding the associations observed in Model I and Model II can be found in Table S6.

### 3.4. Concentration–Response Relationships Linking PM_2.5_ Components to Sleep Duration

As shown in Figure S2, in both 6 mth and 2 yr exposure scenarios, a nonlinear pattern characterized the associations linking PM_2.5_ components with daytime napping duration. Notably, the curves for BC and OM showed a clear initial decline at low concentrations before turning upward, a pattern particularly pronounced in intermediate-term exposure. For the low-concentration range, most of the association curves showed an initial decrease followed by an increase, while some exhibited a direct upward pattern. As the concentration increased, intermediate-term curves exhibited a slight decreasing trend, while long-term curves reached a plateau. As for nocturnal sleep duration, we only observed a nonlinear trend in long-term exposure to NO_3_^−^ (*p* < 0.05).

### 3.5. Stratified Analysis

Notably, intermediate- and long-term exposure scenarios exhibited a comparable pattern for the stratified analysis (Figures S3 and S4). For instance, the effects of 6 mth and 2 yr mean concentrations of SO_4_^2−^ on nocturnal sleep duration were more pronounced among males, smokers, individuals living in rural areas, those who use solid fuel as the primary energy source for cooking and heating, and individuals with chronic diseases and social activities and without pensions. However, the difference remained statistically significant only for residence type in both intermediate- and long-term exposure effects (χ^2^ = 11.243, *p* for interaction = 0.001; χ^2^ = 6.501, *p* for interaction = 0.011), as well as for primary energy source for heating in the intermediate-term association (χ^2^ = 5.211, *p* for interaction = 0.022).

Potential interactions of residence on the relationships were observed in most PM_2.5_ components (*p* for interaction < 0.05), suggesting that living in rural areas may have a more adverse effect on sleep duration. Additionally, heating energy, cooking energy, and pension also influenced the effect of PM_2.5_ components on sleep duration.

### 3.6. Joint Effects of PM_2.5_ Component Mixtures on Sleep Duration

**Figure 3:**
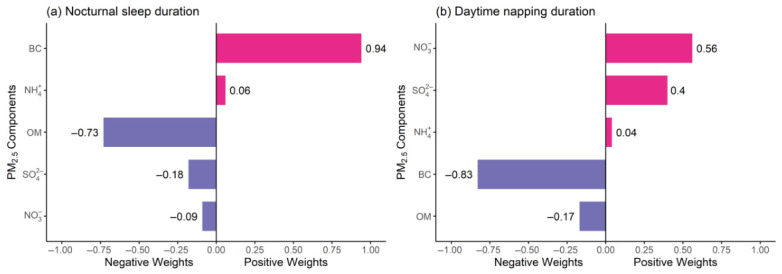
Index weights of five PM_2.5_ components from quantile-based g-computation models for nocturnal sleep and daytime napping duration with per-quartile increases in long-term exposure to PM_2.5_ component mixture.

In the long-term exposure models (Figure 3), per-quartile increases in the PM_2.5_ component mixture were associated with a decrease of 0.04 (β = −0.04, 95% CI: −0.05, −0.02) h in nocturnal sleep duration and an increase of 0.10 (β = 0.10, 95% CI: 0.09, 0.12) h in daytime napping duration. For nocturnal sleep duration, BC had the largest positive weight (0.94), while OM had the largest negative weight (−0.73), suggesting that the overall effect was predominantly driven by OM. Regarding daytime napping duration, NO_3_^−^ and SO_4_^2−^ were the primary contributors with positive weights of 0.56 and 0.40, respectively, while BC showed a negative weight (−0.83).

Results for intermediate-term exposure are presented in Figure S5. Specifically, per-quartile increases in the PM_2.5_ component mixture were associated with a decrease of 0.04 (β = −0.04, 95% CI: −0.05, −0.02) h in nocturnal sleep duration, mainly driven by NH_4_^+^ and OM, and an increase of 0.09 (β = 0.09, 95% CI: 0.08, 0.10) h in daytime napping duration, primarily attributed to NO_3_^−^.

### 3.7. Sensitivity Analysis

Sensitivity analysis confirmed that our main results are robust (Tables S7–S11). For example, the effects of 1 and 3 mth exposure concentrations on sleep duration were similar to the main results of the 6 mth association, while 1 and 3 yr associations remained in line with the main results of the 2 yr association. Excluding individuals with only one visit record, the results of both intermediate- and long-term exposure effects were consistent with our main results.

## 4. Discussion

Non-linear concentration–response relationships were observed in the association between PM_2.5_ components and daytime napping duration. In single-pollutant models, intermediate- and long-term exposure to PM_2.5_ components was negatively associated with nocturnal sleep duration and positively associated with daytime napping duration. In multi-pollutant models, our results showed a significant joint effect of per-quartile increases in PM_2.5_ component mixture on short nocturnal sleep duration, with NH_4_^+^ and OM as the main contributors. In contrast, the mixture was positively associated with extended daytime napping duration, with NO_3_^−^ and SO_4_^2−^ emerging as the primary contributors. Interestingly, effect estimates varied between single-pollutant and multi-pollutant models. Among single-pollutant analyses, BC and SO_4_^2−^ showed the most pronounced change in their association with sleep duration for each IQR increment in concentration. Notably, the observed reductions in nocturnal sleep duration were modest in magnitude (approximately a 4 min per-quartile increase in PM_2.5_ component exposure). However, such small but persistent reductions in sleep duration are considered clinically and public-health-relevant in middle-aged and elderly populations, as cumulative sleep loss over prolonged periods is associated with elevated risks of hypertension [6], diabetes [4] and cardiovascular disease [2]. Even minor chronic sleep disturbances may contribute to substantial health burdens at the population level, supporting the meaningfulness of these associations despite modest individual-level effect sizes.

The divergent associations of PM_2.5_ components with nocturnal sleep and daytime napping can be biologically explained by three established pathways: PM_2.5_-triggered oxidative stress, autonomic nervous system activation, and circadian rhythm dysregulation. Exposure to PM_2.5_ fractions such as OM and NH_4_^+^ induces systemic oxidative stress and low-grade inflammation, which further activates autonomic nervous system imbalance and sympathetic overactivity, thereby disturbing nocturnal sleep maintenance [50,51]. Beyond disrupting redox balance and core clock gene expression, PM_2.5_ exposure also elevates stress hormone metabolites, including 18-oxocortisol and 5α-tetrahydrocortisol, and perturbs circulating circadian biomarkers such as melatonin, retinol, and 5-methoxytryptophol [52]. The combined disruption of redox homeostasis, autonomic modulation, and circadian rhythmicity ultimately impairs the sleep–wake cycle, leading to shortened nocturnal sleep duration and compensatory prolongation of daytime napping.

Notably, in urban environments, BC and nitrogen oxides (NO_x_) are primarily sourced from traffic emissions and are highly correlated with road traffic noise—a well-documented environmental disruptor of sleep. Traffic noise can directly induce sleep fragmentation, reduce deep sleep duration, and alter circadian rhythms via auditory arousal and autonomic nervous system dysregulation [28,53]. Therefore, the observed associations of BC and NO_x_ with adverse sleep outcomes may be partially confounded or synergistically amplified by co-occurring traffic noise, as these two stressors often coexist in densely populated urban areas.

Contemporary epidemiological research has extensively examined how exposure to criteria air pollutants correlates with various sleep disorders [30,39,54,55,56,57,58]. Few have previously discussed how PM_2.5_ components negatively affect the quality or duration of sleep [34,35,59]. The first study found that higher exposure levels of PM_2.5_, BC, SO_4_^2−^, NO_3_^−^, and NH_4_^+^ would lead to poor sleep quality, with each IQR increase in these components corresponding to ORs (95% CI) of 1.335 (1.292, 1.378), 1.124 (1.107, 1.140), 1.097 (1.080, 1.113), 1.137 (1.100, 1.174), and 1.197 (1.156, 1.240), respectively [34], aligning with another study conducted among children and adolescents [60]. However, no significant positive association was observed for OM exposure in the first study. The third study assessed the relationship between long-term exposure to ambient BC and sleep disturbances among college students, reporting significant positive associations across quartiles (Quartile 2: OR = 1.15, 95% CI: 1.04–1.27; Quartile 3: OR = 1.16, 95% CI: 1.04–1.30; Quartile 4: OR = 1.26, 95% CI: 1.11–1.43) compared to the lowest quartile [59]. In contrast to our findings and previous studies [57], a study based on wearable device data reported that exposure to PM_2.5_ and its five components significantly extended total sleep duration [35]. Such discrepancies may be attributed to differences in study populations, data collection methods, study regions, and modeling approaches.

Habitual daytime napping used to be considered a common behavioral and lifestyle practice, yet the existing literature suggests that excessive napping duration may be associated with adverse health outcomes, particularly cardiovascular disease [61], all-cause mortality [62], and an increased risk of metabolic disorders [63,64].

This study found significant non-linear associations of intermediate- and long-term exposure to BC and OM with daytime napping duration (all *p* for non-linearity < 0.001), following a threshold effect pattern: the effect estimates initially declined at low concentrations and then rose after passing the inflection point. At low concentrations, BC exposure may transiently activate the sympathetic nervous system and trigger mild stress responses, temporarily reducing daytime sleepiness [65]. When exposure exceeded a certain threshold, however, sustained oxidative stress and airway inflammation gradually became dominant, leading to increased fatigue and prolonged daytime napping duration [62]. Notably, given the consistent non-linear trends observed, these associations should be interpreted with caution and cannot be considered as stable independent effects.

Our stratified analysis revealed that individuals using solid fuels as their primary energy source for heating experienced stronger detrimental impacts from PM_2.5_ components. In this study, we used fuel types as proxies for indoor pollution levels, and those relying on solid fuels were likely to face higher levels of PM_2.5_ exposure, thereby increasing their susceptibility to sleep duration. Possible explanations for these findings include the incomplete combustion of solid fuels, which generates higher concentrations of harmful pollutants such as particulate matter, carbon monoxide, and polycyclic aromatic hydrocarbons [66].

Similarly, individuals without pensions, representing lower-income groups, were more vulnerable to the adverse effects of PM_2.5_ exposure on sleep duration. This may be explained by the fact that lower-income populations often reside in environments with higher pollution levels, such as areas near major traffic roads or industrial zones, while higher-income individuals tend to have better housing conditions and protective facilities, including advanced ventilation systems and broader residential green spaces [67,68]. Moreover, limited access to healthcare resources among low-income groups exacerbates their health burden from PM_2.5_ exposure. Existing evidence has also suggested that higher income levels can effectively improve health outcomes [69], which aligns with our findings.

Additionally, a statistically significant difference in susceptibility to PM_2.5_ components between rural and urban residents was observed, with rural populations being more affected by PM_2.5_-related sleep duration. This disparity is consistent with previous research [29,70,71] and may be attributed to inequities in air pollution distribution [72] and limited access to healthcare services in rural areas [73]. Furthermore, rural residents, often at lower socioeconomic levels, are subject to greater psychosocial stressors, which may further elevate their risk of sleep problems [74,75]. Musculoskeletal pain, prevalent among manual laborers in rural areas, could also contribute to nocturnal sleep disruption by exacerbating physical discomfort [76].

Strong collinearity was observed among PM_2.5_ components (Pearson r = 0.78–0.99), which may reduce the stability and precision of component-specific weights in qgcomp. This collinearity, arising from shared emission sources and chemical covariation, can alter the magnitude and direction of individual weights compared with single-pollutant models [77]. Notably, single-pollutant models capture unadjusted effects potentially confounded by co-occurring pollutants, whereas qgcomp weights reflect each component’s independent contribution net of other mixture constituents [78]. Although collinearity impacts individual weight precision, it does not invalidate the overall mixture effect, so component-specific weights should be interpreted cautiously. For BC, single-pollutant models showed negative associations with nocturnal sleep duration and positive associations with daytime napping, but qgcomp yielded opposite weights (0.94 for nocturnal sleep, −0.83 for daytime napping). This apparent inconsistency does not represent a genuine reversal of BC’s biological impact, nor does it imply a plausible protective effect. Instead, it is a statistical artifact caused by strong collinearity: when highly correlated components are mutually adjusted within the mixture framework, the model redistributes relative contribution weights mathematically, leading to reversed individual directions that cannot be interpreted as real independent health effects. The major PM_2.5_ components contributing to sleep outcomes varied by exposure window, indicating component-specific and time-dependent mechanisms. For nocturnal sleep duration (inversely associated with all components in single-pollutant models), qgcomp negative weights were dominated by NH_4_^+^ and OM in the 6 mth window (attributable to acute inflammation and oxidative stress disrupting sleep structure) [62] and by OM and SO_4_^2−^ in the 2 yr window (reflecting cumulative cytotoxicity and sustained systemic inflammation) [79]. For daytime napping (positively associated with all components in single-pollutant models), qgcomp positive weights were driven by NO_3_^−^ in the 6 mth window (linked to acute airway irritation and compensatory daytime drowsiness) [40] and jointly by NO_3_^−^ and SO_4_^2−^ in the 2 yr window (consistent with chronic inflammation and vascular impairment causing persistent daytime fatigue) [80]. These patterns are biologically plausible: secondary inorganic aerosols (NO_3_^−^, SO_4_^2−^) elicit acute/chronic inflammation to alter sleep, while carbonaceous components (BC, OM) exert cumulative effects on autonomic and circadian function [81]. BC’s contradictory weights further reflect collinearity-induced mutual adjustment, not genuine biological reversal.

## 5. Strengths and Limitations

Our study has two notable strengths. Firstly, this is the first study to examine the associations between both intermediate- and long-term exposure to PM_2.5_ components and sleep duration, using data from a large, nationwide prospective cohort. Additionally, an outstanding contribution of this study is its pioneering examination of the effects of ambient PM_2.5_ and its components on daytime napping duration, an important sleep parameter.

This study was subject to several potential limitations. First, the concentrations of PM_2.5_ components were assigned at the city level instead of precise residential addresses, which may introduce exposure misclassification. Such spatial averaging obscures within-city variability in pollutant levels and tends to attenuate observed effect sizes toward the null, potentially leading to underestimation of the true associations between long-term PM_2.5_ component exposure and sleep outcomes [82,83]. Second, although we adjusted for a range of potential covariates, some key factors—such as noise, secondhand smoke, dietary habits, and use of sleep medications—could not be accounted for, introducing potential bias and uncertainty into our effect estimates. Notably, traffic noise, which is highly correlated with BC and NO_x_ in urban settings, may further confound the observed associations (see Discussion for detailed elaboration). Third, as this longitudinal study focused on middle-aged and elderly adults, the generalizability of our findings to other populations may be limited. Fourth, nocturnal sleep and daytime napping duration were assessed through self-reported questionnaires, which are subject to recall bias and measurement error. Validation studies [84,85] among middle-aged and elderly Chinese populations have documented moderate but imperfect correlations between self-reported sleep duration and objective measurements (e.g., actigraphy), supporting reasonable validity while highlighting inherent inaccuracy. Furthermore, systematic reporting bias in self-reported sleep duration has been widely reported across socioeconomic, demographic, and geographic subgroups [86,87]. Given that subgroup analyses by these characteristics were conducted in this study, such systematic bias may influence the comparability and interpretation of stratified results. Caution is therefore warranted when interpreting findings across subgroups. Finally, the exposure concentrations of PM_2.5_ components were matched based on the reported addresses at each wave, which could lead to errors if the participants changed their residences.

## 6. Conclusions

Overall, non-linear associations were observed between PM_2.5_ components and daytime napping duration. Our robust findings suggested that both intermediate- and long-term exposure to PM_2.5_ components corresponded with reduced nocturnal sleep and increased daytime napping. Furthermore, multi-pollutant models indicated a significant joint effect of PM_2.5_ mixtures on short nocturnal sleep, primarily driven by NH_4_^+^ and OM, and on prolonged daytime napping, mainly attributable to NO_3_^−^ and SO_4_^2−^. Living in rural areas, the absence of pensions, and usage of solid fuel as the primary energy source for heating may further exacerbate this adverse effect. These findings could provide valuable insights into the effective design of PM_2.5_ control and intervention measures to protect overall sleep health.

## Figures and Tables

**Table 1:** Basic characteristics of survey responses included in the study (*n* = 35,832).

Characteristic		*n* (%)/Median [IQR]
Gender	Male	15,689 (43.8)
	Female	20,143 (56.2)
Age	≥65 years	10,515 (29.3)
	45–65 years	25,317 (70.7)
Smoking *	No	26,545 (78.5)
	Yes	7279 (21.5)
Drinking *	≤1/Month	2854 (8.0)
	>1/Month	8974 (25.0)
Disability	No	26,818 (74.8)
	Yes	9014 (25.2)
Residence	Rural	21,496 (60.0)
	Urban	14,336 (40.0)
Heating *	Clean energy	5228 (33.5)
	Solid fuel	10,357 (66.5)
Cooking *	Clean energy	21,477 (60.1)
	Solid fuel	14,261 (39.9)
Marital status	Divorced/widowed/separated	3999 (11.2)
	Married/cohabiting	31,644 (88.3)
	Unmarried	189 (0.5)
Education	Low	22,752 (63.5)
	Median	12,310 (34.4)
	High	770 (2.1)
Chronic diseases	No	13,686 (38.2)
	Yes	22,146 (61.8)
Pension	No	30,396 (84.8)
	Yes	5436 (15.2)
Social activities	No	15,964 (44.6)
	Yes	19,868 (55.4)

* Some data is missing.

## Data Availability

The data used in this study were obtained from the publicly available China Health and Retirement Longitudinal Study (CHARLS). Restrictions apply to the availability of these data, which were used under license for this study. Data can be accessed by qualified researchers through the official CHARLS website (http://charls.pku.edu.cn/, accessed on 6 March 2025).

## Supplementary Materials

The following supporting information can be downloaded at: https://www.mdpi.com/article/10.3390/toxics14050437/s1, Figure S1: Flow chart of the study sample; Figure S2. Association between intermediate- and long-term exposure to PM_2.5_ components and nocturnal sleep and daytime napping duration using a restricted cubic spline regression model. The solid lines represent estimates of the effects, and the shadowed areas represent the 95% confidence intervals (CIs); Figure S3. Stratified analysis of β with 95% confidence intervals (CIs) in nocturnal sleep duration per IQR increment in the intermediate- and long-term average concentrations of PM_2.5_ and its chemical components; Figure S4. Stratified analysis of changes with 95% confidence intervals (CIs) in daytime napping duration per IQR increment in the intermediate- and long-term mean concentrations of PM_2.5_ and its chemical components; Figure S5. Index weights of five PM_2.5_ components from quantile-based g-computation models for nocturnal sleep and daytime napping duration with per-quartile increases in intermediate-term exposure to PM_2.5_ component mixture; Table S1. The definition of covariates in this study; Table S2. Statistical description of PM_2.5_ components and meteorological parameters; Table S3. Pearson’s correlations of intermediate-term PM_2.5_ and its chemical components; Table S4. Pearson’s correlations of long-term PM_2.5_ and its chemical components; Table S5. Associations between intermediate- and long-term exposure to PM_2.5_ components (per IQR increase) and nocturnal sleep duration; Table S6. Associations between intermediate- and long-term exposure to PM_2.5_ components and daytime napping duration across quartile levels based on Models I and II; Table S7. Sensitivity analysis of associations between PM_2.5_ components (per IQR increase) and nocturnal sleep duration based on different exposure windows; Table S8. Sensitivity analysis of associations between PM_2.5_ components and daytime napping duration based on different exposure windows; Table S9. Sensitivity analysis of associations between PM_2.5_ components (per IQR increase) and nocturnal sleep duration in two groups; Table S10. Sensitivity analysis of associations between PM_2.5_ components and daytime napping duration based on Model III in two groups; Table S11. Sensitivity analysis of the associations between intermediate- and long-term exposure to PM_2.5_ components and nocturnal sleep duration across quartile levels based on Model III.

## Abbreviations

The following abbreviations are used in this manuscript:

CHARLS	China Health and Retirement Longitudinal Study
Qgcomp	Quantile-based g-computation
PM_2.5_	Fine particulate matter
BC	Black carbon
OM	Organic matter
SO_4_^2−^	Sulfate
NO_3_^−^	Nitrate
NH_4_^+^	Ammonium
IQR	Interquartile range
RCS	Restricted cubic spline
β	Coefficient
CI	Confidence interval
NO_x_	Nitrogen oxides
