# Supplementary material for: Intermediate- and Long-Term Exposure to PM2.5 and Its Chemical Components in Relation to Nocturnal Sleep Duration and Daytime Napping Duration
Source: Toxics. 2026 May 14;14(5):437. doi: 10.3390/toxics14050437 (PMC13211739; doi:10.3390/toxics14050437)
Supplement: Supplementary file 1 [file toxics-14-00437-s001.zip › toxics-4281839-supplementary.pdf]

Supplement of

Intermediate- and Long-Term Exposure to $PM_{2.5}$ and Its Chemical Components in Relation to Nocturnal Sleep Duration and Daytime Napping Duration

Figure S1. Flow chart of the study sample.

Figure S2. Association between intermediate- and long-term exposure to $PM_{2.5}$ components and nocturnal sleep and daytime napping duration using a restricted cubic spline regression model. The solid lines represent estimates of the effects, and the shadowed areas represent the 95% confidence intervals (CIs).

Figure S3. Stratified analysis of β with 95% confidence intervals (CIs) in nocturnal sleep duration per IQR increment in the intermediate- and long-term average concentrations of $PM_{2.5}$ and its chemical components.

Figure S4. Stratified analysis of changes with 95% confidence intervals (CIs) in daytime napping duration per IQR increment in the intermediate- and long-term mean concentrations of $PM_{2.5}$ and its chemical components.

Figure S5. Index weights of five $PM_{2.5}$ components from quantile-based g-computation models for nocturnal sleep and daytime napping duration with per quartile increases in the intermediate-term exposure of $PM_{2.5}$ component mixture.

Table S1. The definition of covariates in this study.

Table S2. Statistical descriptive of $PM_{2.5}$ components and meteorological parameters.

Table S3. Pearson's correlations of intermediate-term $PM_{2.5}$ and its chemical components.

Table S4. Pearson's correlations of long-term $PM_{2.5}$ and its chemical components.

Table S5. Associations between intermediate- and long-term exposure to $PM_{2.5}$ components (per IQR increase) and nocturnal sleep duration.

Table S6. Associations between intermediate- and long-term exposure to $PM_{2.5}$ components and daytime napping duration across quartile levels based on model I and II.

Table S7. Sensitivity analysis of associations between $PM_{2.5}$ components (per IQR increase) and nocturnal sleep duration based on different exposure windows.

Table S8. Sensitivity analysis of associations between $PM_{2.5}$ components and daytime napping duration based on different exposure windows.

Table S9. Sensitivity analysis of associations between $PM_{2.5}$ components (per IQR increase) andnocturnal

sleep duration in two groups.

Table S10. Sensitivity analysis of associations between $PM_{2.5}$ components and daytime napping duration based on model III in two groups.

Table S11. Sensitivity analysis of the associations between intermediate- and long-term exposure to $PM_{2.5}$ components and nocturnal sleep duration across quartile levels based on model III.

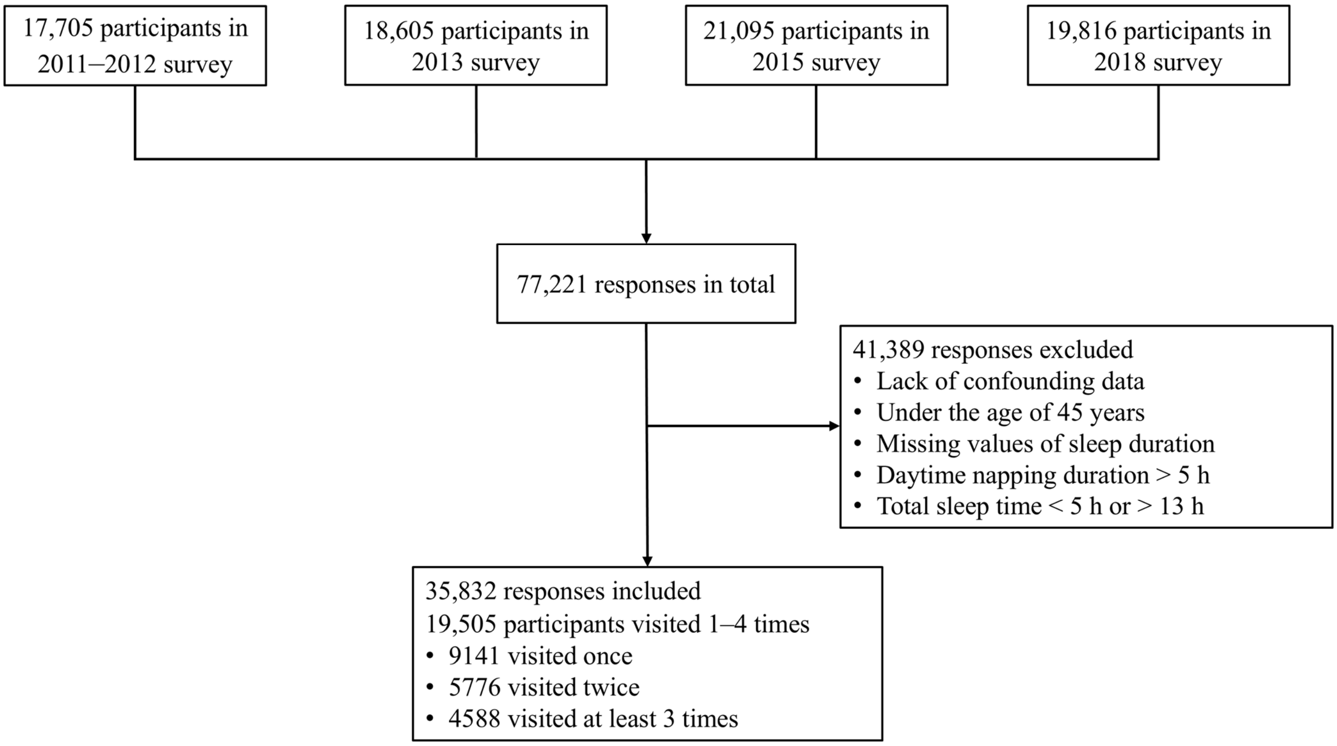

Figure S1. Flow chart of the study sample.

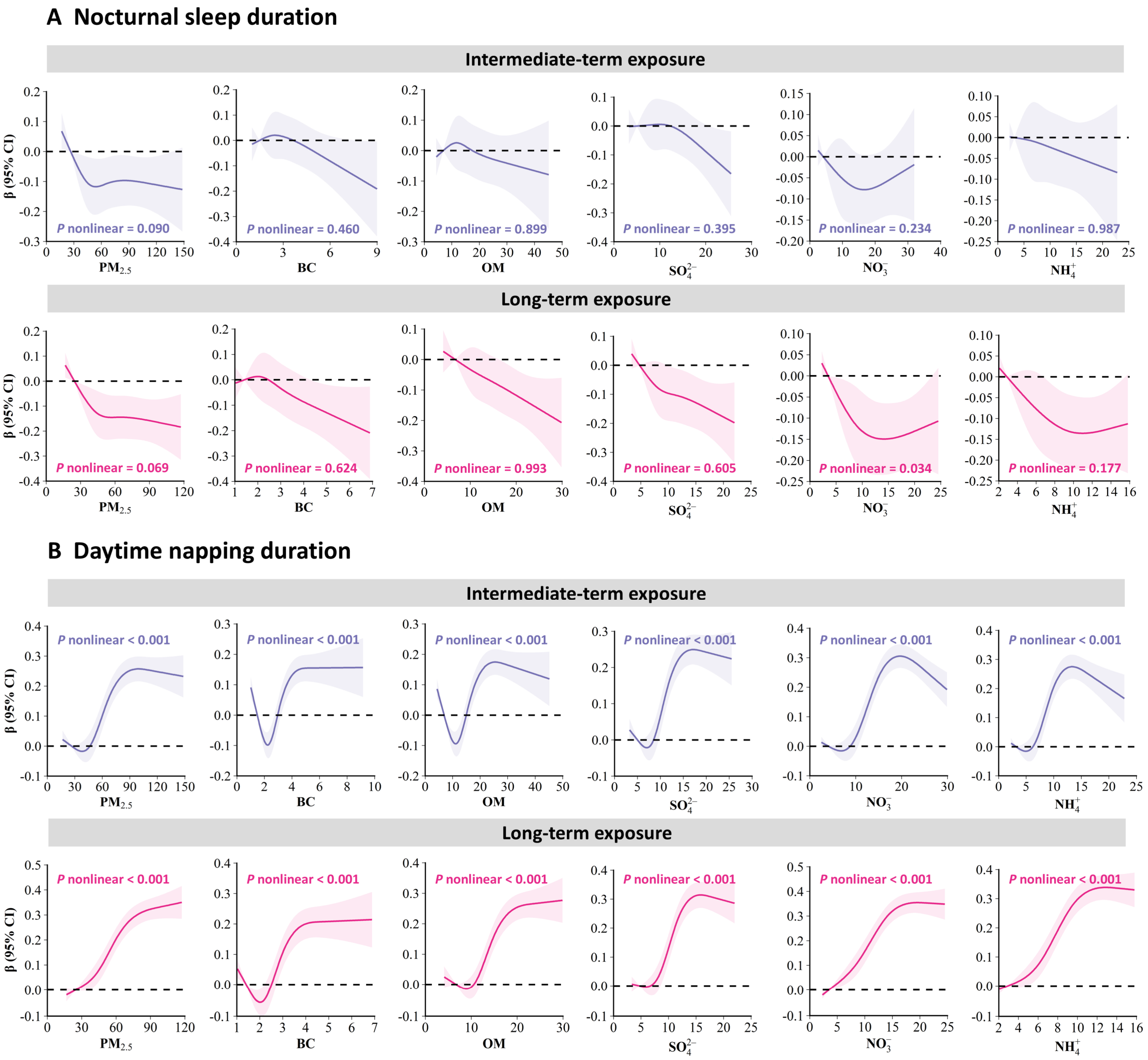

Figure S2. Association between intermediate- and long-term exposure to $PM_{2.5}$ components and nocturnal sleep and daytime napping duration using a restricted cubic spline regression model. The solid lines represent estimates of the effects, and the shadowed areas represent the 95% confidence intervals (CIs).

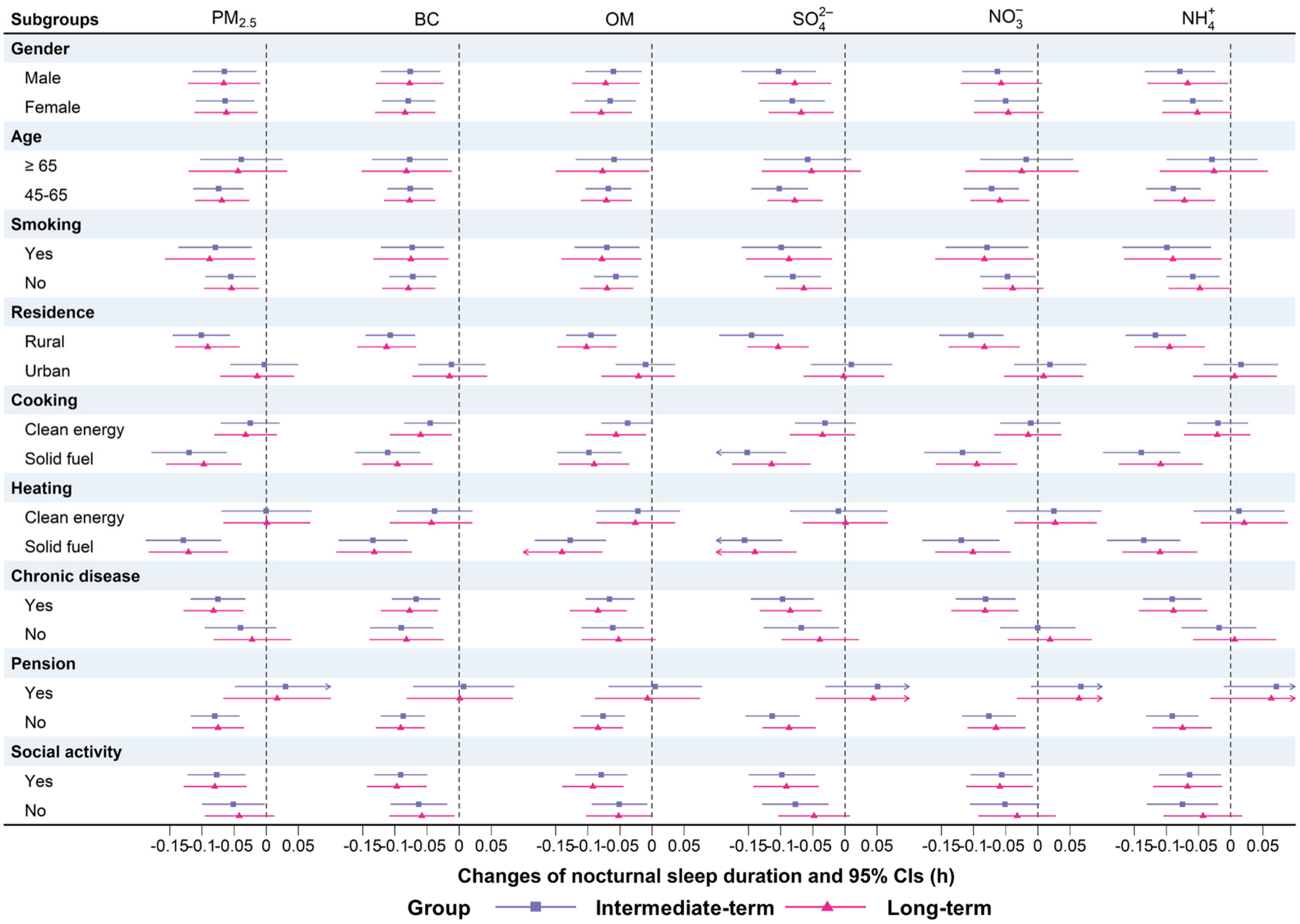

Figure S3. Stratified analysis of β with 95% confidence intervals (CIs) in nocturnal sleep duration per IQR increment in the intermediate- and long-term average concentrations of $PM_{2.5}$ and its chemical components.

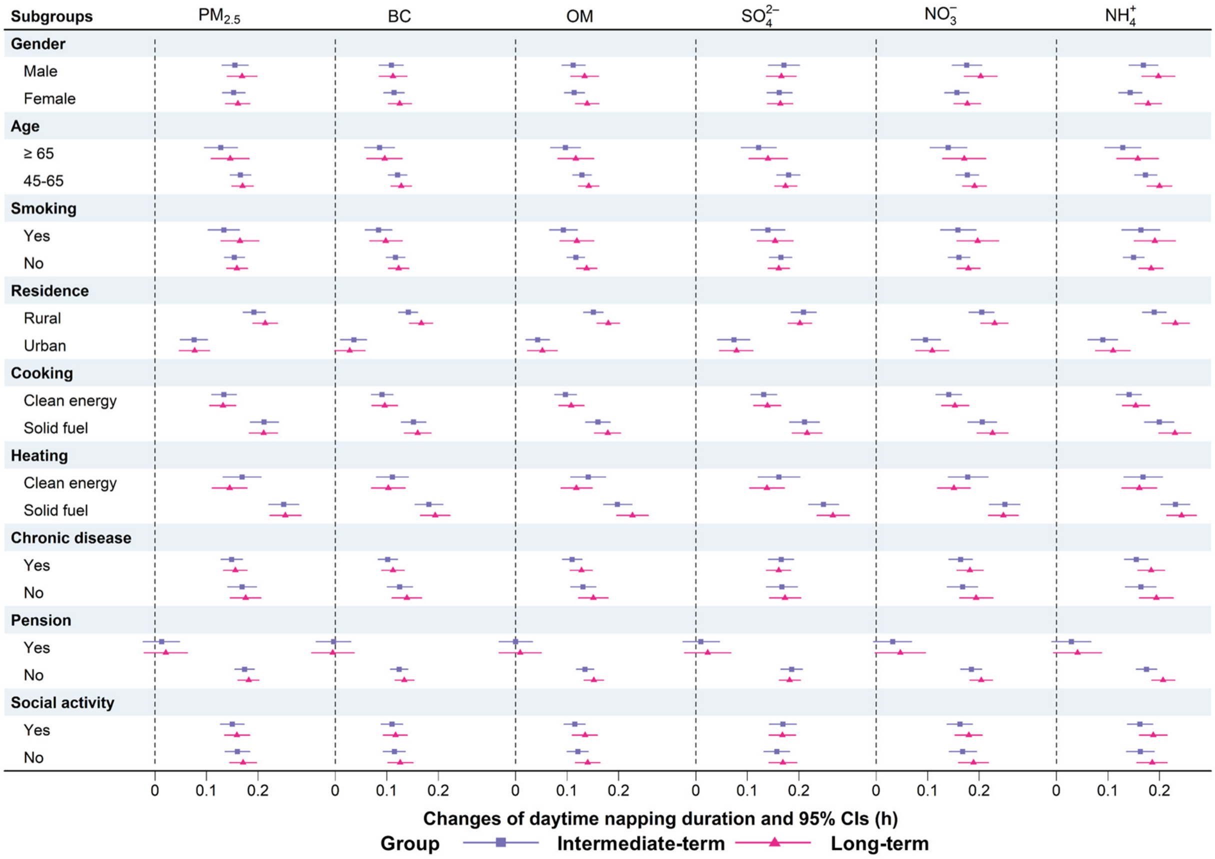

Figure S4. Stratified analysis of changes with 95% confidence intervals (CIs) in daytime napping duration per IQR increment in the intermediate- and long-term mean concentrations of $PM_{2.5}$ and its chemical components.

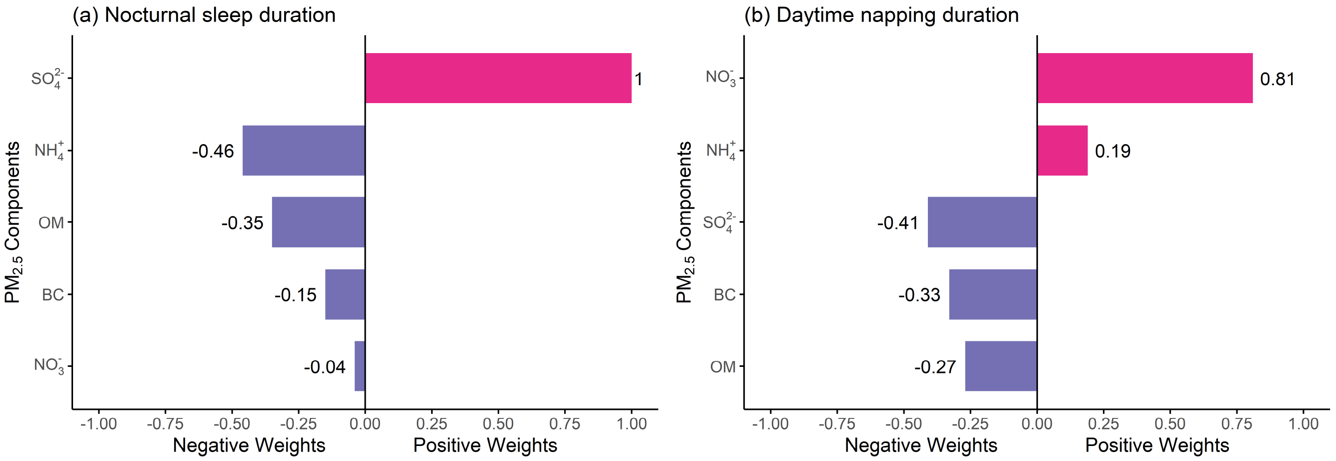

Figure S5. Index weights of five $PM_{2.5}$ components from quantile-based g-computation models for nocturnal sleep and daytime napping duration with per quartile increases in the intermediate-term exposure of $PM_{2.5}$ component mixture.

Table S1. The definition of covariates in this study.

Type of deficit	Item	Variables	Definition
Demographic characteristics	1	Age	1 = 45-65 years; 2= ≥ 65 years
	2	Gender	1 = Male; 2 = Female
	3	Marital status	1 = Divorced/widowed/separated;<br>2 = Married/cohabiting;<br>3 = Unmarried
	4	Residence	1 = Rural; 2 = Urban
	5	Education level	1 = Primary school or below;<br>2 = Middle school/high school/vocational school;<br>3 = College or above
	6	Pension	1 = Yes; 0 = No
Lifestyle characteristics	7	Smoking	1 = Current smoking; 0 = Never smoked or formerly smoked
	8	Drinking	0 = No drinking;<br>1 = Drink but less than once a month;<br>2 = Drink more than once a month
	9	Disability	0 = No; 1 = Yes
	10	Chronic diseases	**Contents:** Hypertension, dyslipidemia, diabetes, cancer, chronic lung diseases, liver disease, heart disease, stroke, kidney disease, digestive diseases, emotional problems, memory-related disease, arthritis, and asthma<br>0 = No; 1 = Yes
	11	Social activities	**Contents:** Interacted with friends; played Ma-jong, played chess, played cards, or went to community club; provided help; went to a sport, social, or other kind of club; took part in a community-related organization; done voluntary or charity work; cared for a sick or disabled adult; attended an educational or training course; stock investment; used the internet; other<br>0 = No; 1 = Yes
	12	Cooking	1 = Clean energy; 2 = Solid fuel
	13	Heating	1 = Clean energy; 2 = Solid fuel
Meteorological variables	14	Temperature	Mean temperature, °C
	15	Relative humidity	Mean relative humidity, %
	16	Wind speed	Mean wind speed, m/s

Table S2. Statistical descriptive of $PM_{2.5}$ components and meteorological parameters.

Exposure	Variables	Summary statistics							
		Mean	SD	Min	P_{25}	Median	P_{75}	Max	IQR
Intermediate-term	$PM_{2.5}$ (μg/m^3)	61.86	26.97	16.57	41.10	57.38	77.60	148.23	36.50
	BC (μg/m^3)	2.98	1.23	1.01	2.03	2.74	3.56	9.16	1.53
	OM (μg/m^3)	15.43	6.79	4.32	10.42	14.06	19.09	45.16	8.67
	SO_4^{2-} (μg/m^3)	10.73	4.39	3.28	7.12	9.92	13.61	25.53	6.49
	NO_3^- (μg/m^3)	13.05	6.38	2.52	7.80	12.69	17.35	31.95	9.55
	NH_4^+ (μg/m^3)	8.69	3.98	2.25	5.58	8.28	11.29	22.81	5.71
	Temperature (°C)	11.70	6.17	-15.32	8.55	13.00	15.85	22.82	7.30
	Relative humidity (%)	59.56	8.15	37.22	54.39	59.94	65.61	77.06	11.21
	Wind speed (m/s)	1.40	0.44	0.31	1.13	1.36	1.66	2.94	0.54
Long-term	$PM_{2.5}$ (μg/m^3)	56.46	22.58	16.65	39.35	53.20	71.72	117.35	32.37
	BC (μg/m^3)	2.78	1.01	1.03	2.01	2.60	3.34	6.89	1.33
	OM (μg/m^3)	13.66	5.21	4.10	9.78	12.76	16.73	30.13	6.95
	SO_4^{2-} (μg/m^3)	10.38	4.01	3.31	7.10	9.89	12.96	22.00	5.85
	NO_3^- (μg/m^3)	11.55	5.32	2.28	7.09	11.27	15.48	24.53	8.39
	NH_4^+ (μg/m^3)	7.91	3.37	2.06	5.16	7.63	10.49	15.80	5.33
	Temperature (°C)	14.99	48.84	-1.86	13.37	15.95	18.02	23.83	46.50
	Relative humidity (%)	62.60	6.98	43.03	57.73	63.28	67.88	75.81	10.15
	Wind speed (m/s)	1.37	0.37	0.31	1.11	1.35	1.61	2.48	0.49

Abbreviations: Min, minimum; Max, maximum; SD, standard deviation; P_{25}, 25th percentile; P_{75}, 75th percentile; IQR, interquartile range.

Table S3. Pearson's correlations of intermediate-term $PM_{2.5}$ and its chemical components.

	$PM_{2.5}$	BC	OM	SO_4^{2-}	NO_3^-	NH_4^+
$PM_{2.5}$	1.00	0.94***	0.96***	0.95***	0.94***	0.92***
BC		1.00	0.98***	0.92***	0.81***	0.82***
OM			1.00	0.90***	0.84***	0.83***
SO_4^{2-}				1.00	0.94***	0.95***
NO_3^-					1.00	0.99***
NH_4^+						1.00

Notes: *** The *P* value for the correlation is < 0.001.

Table S4. Pearson's correlations of long-term $PM_{2.5}$ and its chemical components.

	$PM_{2.5}$	BC	OM	SO_4^{2-}	NO_3^-	NH_4^+
$PM_{2.5}$	1.00	0.93***	0.97***	0.96***	0.94***	0.94***
BC		1.00	0.97***	0.94***	0.78***	0.82***
OM			1.00	0.93***	0.86***	0.87***
SO_4^{2-}				1.00	0.92***	0.95***
NO_3^-					1.00	0.99***
NH_4^+						1.00

Notes: *** The P value for the correlation is < 0.001.

Table S5. Associations between intermediate- and long-term exposure to $PM_{2.5}$ components (per IQR increase) and nocturnal sleep duration.

	Intermediate-term exposure, β (95% CIs)			Long-term exposure, β (95% CIs)		
	Model I	Model II	Model III	Model I	Model II	Model III
$PM_{2.5}$	-0.08 (-0.10, -0.05)	-0.07 (-0.10, -0.05)	-0.07 (-0.10, -0.04)	-0.08 (-0.10, -0.05)	-0.07 (-0.10, -0.04)	-0.07 (-0.10, -0.03)
BC	-0.08 (-0.11, -0.06)	-0.08 (-0.11, -0.06)	-0.08 (-0.11, -0.05)	-0.08 (-0.11, -0.06)	-0.08 (-0.11, -0.05)	-0.08 (-0.12, -0.05)
OM	-0.07 (-0.09, -0.05)	-0.07 (-0.09, -0.05)	-0.07 (-0.09, -0.04)	-0.08 (-0.11, -0.06)	-0.08 (-0.11, -0.05)	-0.08 (-0.11, -0.04)
SO_4^{2-}	-0.09 (-0.12, -0.06)	-0.10 (-0.13, -0.06)	-0.10 (-0.13, -0.06)	-0.09 (-0.11, -0.06)	-0.08 (-0.11, -0.06)	-0.07 (-0.11, -0.04)
NO_3^-	-0.07 (-0.10, -0.04)	-0.07 (-0.10, -0.05)	-0.06 (-0.10, -0.02)	-0.07 (-0.10, -0.05)	-0.07 (-0.10, -0.04)	-0.05 (-0.09, -0.01)
NH_4^+	-0.07 (-0.10, -0.05)	-0.08 (-0.11, -0.05)	-0.07 (-0.11, -0.04)	-0.08 (-0.11, -0.05)	-0.08 (-0.11, -0.05)	-0.06 (-0.10, -0.02)

Table S6. Associations between intermediate- and long-term exposure to $PM_{2.5}$ components and daytime napping duration across quartile levels based on model I and II.

	Intermediate-term exposure (6-mth)				Long-term exposure (2-yr)			
	Model I		Model II		Model I		Model II	
	β	95% CIs	*β*	95% CIs	*β*	95% CIs	*β*	95% CIs
$PM_{2.5}$								
Q1	Reference		Reference		Reference		Reference	
Q2	0.10	0.08, 0.12***	0.09	0.07, 0.11***	0.10	0.07, 0.12***	0.09	0.07, 0.11***
Q3	0.22	0.20, 0.24***	0.22	0.20, 0.24***	0.22	0.20, 0.25***	0.22	0.20, 0.24***
Q4	0.33	0.30, 0.35***	0.32	0.30, 0.35***	0.30	0.28, 0.33***	0.30	0.27, 0.32***
BC								
Q1	Reference		Reference		Reference		Reference	
Q2	0.07	0.05, 0.09***	0.07	0.05, 0.09***	0.10	0.08, 0.13***	0.10	0.08, 0.12***
Q3	0.17	0.15, 0.20***	0.17	0.14, 0.19***	0.19	0.16, 0.21***	0.18	0.16, 0.21***
Q4	0.27	0.24, 0.30***	0.27	0.24, 0.30***	0.29	0.27, 0.32***	0.29	0.27, 0.32***
OM								
Q1	Reference		Reference		Reference		Reference	
Q2	0.08	0.06, 0.10***	0.08	0.05, 0.10***	0.12	0.10, 0.14***	0.11	0.09, 0.14***
Q3	0.19	0.17, 0.21***	0.19	0.17, 0.21***	0.20	0.17, 0.22***	0.19	0.17, 0.22***
Q4	0.28	0.25, 0.31***	0.28	0.25, 0.31***	0.30	0.27, 0.32***	0.29	0.27, 0.32***
SO_4^{2-}								
Q1	Reference		Reference		Reference		Reference	
Q2	0.10	0.07, 0.12***	0.09	0.07, 0.11***	0.10	0.08, 0.12***	0.09	0.07, 0.12***
Q3	0.21	0.19, 0.23***	0.20	0.18, 0.23***	0.22	0.20, 0.25***	0.21	0.19, 0.24***
Q4	0.30	0.27, 0.33***	0.29	0.26, 0.32***	0.31	0.28, 0.33***	0.30	0.27, 0.33***
NO_3^-								
Q1	Reference		Reference		Reference		Reference	
Q2	0.07	0.05, 0.09***	0.06	0.04, 0.09***	0.10	0.08, 0.13***	0.10	0.08, 0.13***
Q3	0.23	0.21, 0.25***	0.23	0.20, 0.25***	0.22	0.20, 0.24***	0.22	0.19, 0.24***
Q4	0.29	0.26, 0.31***	0.28	0.25, 0.31***	0.29	0.27, 0.32***	0.29	0.26, 0.31***
NH_4^+								
Q1	Reference		Reference		Reference		Reference	
Q2	0.08	0.06, 0.10***	0.07	0.05, 0.10***	0.09	0.07, 0.12***	0.09	0.07, 0.11***
Q3	0.21	0.19, 0.23***	0.21	0.18, 0.23***	0.22	0.20, 0.25***	0.22	0.20, 0.24***
Q4	0.29	0.26, 0.31***	0.28	0.25, 0.30***	0.29	0.27, 0.32***	0.29	0.26, 0.32***

Abbreviations: Q1, below the 25th percentile of air pollutant concentrations (the reference group); Q2, the 25th to 50th percentile of air pollutant concentrations; Q3, the 50th to 75th percentile of air pollutant concentrations; Q4, above 75th percentile of air pollutant concentrations. Notes: *** The *P* value for the confidence interval is < 0.001.

Table S7. Sensitivity analysis of associations between $PM_{2.5}$ components (per IQR increase) and nocturnal sleep duration based on different exposure windows.

	Nocturnal sleep duration, β (95% CIs)			
	1-mth	3-mth	1-yr	3-yr
$PM_{2.5}$	-0.35 (-0.70, -0.01)	-0.48 (-0.79, -0.18)	-0.59 (-0.93, -0.24)	-0.68 (-1.08, -0.28)
BC	-0.73 (-1.09, -0.36)	-0.74 (-1.05, -0.43)	-0.75 (-1.08, -0.42)	-0.89 (-1.27, -0.52)
OM	-0.61 (-0.99, -0.23)	-0.48 (-0.75, -0.22)	-0.69 (-1.03, -0.36)	-0.81 (-1.18, -0.43)
SO_4^{2-}	-0.48 (-0.85, -0.10)	-0.83 (-1.23, -0.43)	-0.77 (-1.17, -0.37)	-0.79 (-1.21, -0.37)
NO_3^-	-0.19 (-0.51, 0.14)	-0.35 (-0.66, -0.04)	-0.49 (-0.90, -0.09)	-0.47 (-0.89, -0.06)
NH_4^+	-0.28 (-0.62, 0.06)	-0.57 (-0.91, -0.22)	-0.58 (-0.98, -0.18)	-0.58 (-1.00, -0.15)

Table S8. Sensitivity analysis of associations between $PM_{2.5}$ components and daytime napping duration based on different exposure windows.

	1-mth (*β*，95% CI)	3-mth (*β*，95% CI)	1-yr (*β*，95% CI)	3-yr (*β*，95% CI)
$PM_{2.5}$				
Q1	Reference	Reference	Reference	Reference
Q2	0.06 (0.02, 0.11)**	0.08 (0.03, 0.12)***	0.05 (0.01, 0.09)*	0.04 (0.00, 0.08)*
Q3	0.18 (0.13, 0.22)***	0.21 (0.17, 0.26)***	0.20 (0.16, 0.24)***	0.21 (0.17, 0.25)***
Q4	0.32 (0.27, 0.37)***	0.35 (0.30, 0.39)***	0.31 (0.27, 0.35)***	0.31 (0.27, 0.35)***
BC				
Q1	Reference	Reference	Reference	Reference
Q2	0.09 (0.05, 0.14)***	0.16 (0.11, 0.21)***	0.05 (0.01, 0.09)*	0.04 (0.00, 0.08)
Q3	0.08 (0.03, 0.13)**	0.14 (0.09, 0.19)***	0.18 (0.13, 0.23)***	0.16 (0.11, 0.20)***
Q4	0.18 (0.13, 0.23)***	0.35 (0.29, 0.40)***	0.29 (0.24, 0.34)***	0.29 (0.24, 0.33)***
OM				
Q1	Reference	Reference	Reference	Reference
Q2	0.00 (-0.05, 0.05)	0.06 (0.01, 0.11)*	0.11 (0.07, 0.15)***	0.09 (0.05, 0.13)***
Q3	0.05 (0.01, 0.10)*	0.18 (0.13, 0.22)***	0.15 (0.11, 0.19)***	0.21 (0.16, 0.25)***
Q4	0.19 (0.15, 0.24)***	0.31 (0.26, 0.36)***	0.30 (0.26, 0.34)***	0.29 (0.25, 0.33)***
SO_4^{2-}				
Q1	Reference	Reference	Reference	Reference
Q2	0.01 (-0.04, 0.05)	0.05 (0, 0.10)*	0.12 (0.08, 0.17)***	0.12 (0.09, 0.16)***
Q3	0.14 (0.09, 0.19)***	0.19 (0.14, 0.23)***	0.26 (0.22, 0.31)***	0.30 (0.26, 0.34)***
Q4	0.24 (0.19, 0.29)***	0.34 (0.29, 0.39)***	0.35 (0.31, 0.39)***	0.35 (0.31, 0.39)***
NO_3^-				
Q1	Reference	Reference	Reference	Reference
Q2	-0.01 (-0.05, 0.03)	0.04 (0, 0.08)	0.11 (0.08, 0.15)***	0.15 (0.11, 0.18)***
Q3	0.09 (0.05, 0.14)***	0.21 (0.16, 0.25)***	0.24 (0.20, 0.28)***	0.28 (0.24, 0.32)***
Q4	0.24 (0.20, 0.28)***	0.31 (0.27, 0.35)***	0.35 (0.31, 0.39)***	0.36 (0.32, 0.40)***
NH_4^+				
Q1	Reference	Reference	Reference	Reference
Q2	-0.01 (-0.06, 0.03)	0.05 (0.00, 0.09)*	0.12 (0.08, 0.15)***	0.13 (0.09, 0.16)***
Q3	0.09 (0.05, 0.14)***	0.21 (0.17, 0.25)***	0.22 (0.18, 0.26)***	0.26 (0.22, 0.30)***
Q4	0.25 (0.20, 0.29)***	0.34 (0.29, 0.38)***	0.34 (0.31, 0.38)***	0.35 (0.31, 0.39)***

Note: *** the *P* value for the confidence interval is < 0.001, ** the *P* value for the confidence interval is < 0.01, * the *P* value for the confidence interval is < 0.05.

Table S9. Sensitivity analysis of associations between $PM_{2.5}$ components (per IQR increase) and nocturnal sleep duration in two groups.

	Nocturnal sleep duration, β (95% CIs)	
	Group 1	Group 2
$PM_{2.5}$		
6-mth	-0.08 (-0.11, -0.04)*	-0.06 (-0.09, -0.02)*
2-yr	-0.07 (-0.11, -0.03)*	-0.05 (-0.09, -0.01)*
BC		
6-mth	-0.09 (-0.13, -0.06)*	-0.08 (-0.11, -0.04)*
2-yr	-0.09 (-0.14, -0.05)*	-0.08 (-0.12, -0.04)*
OM		
6-mth	-0.08 (-0.11, -0.04)*	-0.06 (-0.10, -0.03)*
2-yr	-0.08 (-0.12, -0.04)*	-0.07 (-0.11, -0.03)*
SO_4^{2-}		
6-mth	-0.11 (-0.15, -0.06)*	-0.08 (-0.12, -0.04)*
2-yr	-0.08 (-0.13, -0.04)*	-0.06 (-0.10, -0.02)*
NO_3^-		
6-mth	-0.06 (-0.10, -0.02)*	-0.04 (-0.08, -0.001)*
2-yr	-0.05 (-0.09, 0.001)*	-0.03 (-0.08, 0.01)*
NH_4^+		
6-mth	-0.07 (-0.11, -0.03)*	-0.05 (-0.09, -0.01)*
2-yr	-0.06 (-0.10, -0.01)*	-0.04 (-0.09, 0.002)*

Group 1: excluded individuals with only one visit record

Group 2: excluded smokers

Notes: * the P value for the confidence interval is < 0.05.

Table S10. Sensitivity analysis of associations between $PM_{2.5}$ components and daytime napping duration based on model III in two groups.

	Intermediate-term exposure (6-mth)				Long-term exposure (2-yr)			
	Group 1		Group 2		Group 1		Group 2	
	β	95% CIs	*β*	95% CIs	*β*	95% CIs	*β*	95% CIs
$PM_{2.5}$								
Q1	Reference		Reference		Reference		Reference	
Q2	0.10	0.06, 0.15***	0.08	0.04, 0.13***	0.08	0.03, 0.12**	0.05	0.01, 0.10*
Q3	0.21	0.17, 0.26***	0.20	0.16, 0.25***	0.22	0.18, 0.27***	0.19	0.15, 0.24***
Q4	0.37	0.32, 0.41***	0.35	0.30, 0.39***	0.32	0.28, 0.36***	0.30	0.26, 0.35***
BC								
Q1	Reference		Reference		Reference		Reference	
Q2	0.02	-0.04, 0.07	0.03	-0.02, 0.08	0.08	0.03, 0.13**	0.07	0.02, 0.12**
Q3	0.17	0.12, 0.22***	0.17	0.12, 0.22***	0.19	0.14, 0.25***	0.19	0.13, 0.24***
Q4	0.30	0.25, 0.35***	0.30	0.25, 0.35***	0.31	0.26, 0.37***	0.29	0.24, 0.34***
OM								
Q1	Reference		Reference		Reference		Reference	
Q2	0.05	0, 0.10*	0.07	0.02, 0.12**	0.14	0.09, 0.18***	0.12	0.07, 0.16***
Q3	0.16	0.11, 0.20***	0.16	0.11, 0.21***	0.17	0.12, 0.22***	0.16	0.11, 0.20***
Q4	0.30	0.25, 0.35***	0.30	0.25, 0.35***	0.32	0.28, 0.37***	0.30	0.25, 0.34***
SO_4^{2-}								
Q1	Reference		Reference		Reference		Reference	
Q2	0.12	0.07, 0.16***	0.14	0.09, 0.18***	0.14	0.09, 0.19***	0.13	0.09, 0.18***
Q3	0.26	0.21, 0.31***	0.27	0.22, 0.33***	0.27	0.22, 0.31***	0.27	0.22, 0.32***
Q4	0.36	0.32, 0.41***	0.37	0.32, 0.42***	0.36	0.32, 0.41***	0.35	0.30, 0.39***
NO_3^-								
Q1	Reference		Reference		Reference		Reference	
Q2	0.03	-0.02, 0.07	0.02	-0.02, 0.06	0.13	0.09, 0.17***	0.10	0.05, 0.14***
Q3	0.25	0.21, 0.30***	0.25	0.21, 0.30***	0.24	0.19, 0.28***	0.22	0.18, 0.27***
Q4	0.32	0.28, 0.37***	0.31	0.27, 0.36***	0.36	0.32, 0.40***	0.33	0.29, 0.38***
NH_4^+								
Q1	Reference		Reference		Reference		Reference	
Q2	0.06	0.02, 0.11**	0.05	0.01, 0.10*	0.13	0.09, 0.18***	0.10	0.06, 0.14***
Q3	0.24	0.20, 0.29***	0.24	0.19, 0.28***	0.24	0.19, 0.28***	0.22	0.17, 0.26***
Q4	0.33	0.29, 0.37***	0.32	0.28, 0.36***	0.36	0.31, 0.40***	0.33	0.28, 0.37***

Group 1: excluded individuals with only one visit record
Group 2: excluded smokers

Table S11. Sensitivity analysis of the associations between intermediate- and long-term exposure to $PM_{2.5}$ components and nocturnal sleep duration across quartile levels based on model III.

	Intermediate-term exposure (6-mth)			Long-term exposure (2-yr)		
	β	95% CI	*P* value	*β*	95% CI	*P* value
$PM_{2.5}$						
Q1	Reference			Reference		
Q2	-0.13	-0.21, -0.04	0.002	-0.08	-0.16, 0.0003	0.041
Q3	-0.14	-0.23, -0.06	<0.001	-0.08	-0.16, -0.003	0.526
Q4	-0.18	-0.26, -0.10	<0.001	-0.15	-0.23, -0.07	<0.001
BC						
Q1	Reference			Reference		
Q2	0.02	-0.07, 0.11	0.678	0.05	-0.04, 0.14	0.300
Q3	-0.07	-0.16, 0.02	0.128	-0.13	-0.22, -0.03	0.007
Q4	-0.13	-0.22, -0.03	0.006	-0.13	-0.22, -0.03	0.006
OM						
Q1	Reference			Reference		
Q2	-0.03	-0.11, 0.06	0.515	-0.10	-0.18, -0.02	0.012
Q3	-0.07	-0.15, 0.02	0.113	-0.09	-0.17, -0.01	0.036
Q4	-0.14	-0.22, -0.05	0.001	-0.18	-0.25, -0.10	<0.001
SO_4^{2-}						
Q1	Reference			Reference		
Q2	0.08	0.002, 0.16	0.068	-0.16	-0.24, -0.08	<0.001
Q3	-0.08	-0.16, 0.02	0.098	-0.13	-0.21, -0.04	0.004
Q4	-0.09	-0.18, -0.003	0.035	-0.18	-0.26, -0.10	<0.001
NO_3^-						
Q1	Reference			Reference		
Q2	-0.01	-0.09, 0.07	0.811	-0.11	-0.19, -0.04	0.003
Q3	-0.08	-0.16, -0.001	0.049	-0.12	-0.20, -0.04	0.004
Q4	-0.12	-0.19, -0.04	0.003	-0.11	-0.19, -0.04	0.003
NH_4^+						
Q1	Reference			Reference		
Q2	0	-0.08, 0.08	0.966	-0.12	-0.19, -0.04	0.003
Q3	-0.10	-0.18, -0.01	0.020	-0.08	-0.16,-0.004	0.051
Q4	-0.12	-0.20, -0.04	0.002	-0.13	-0.21, -0.06	<0.001

Abbreviations: Q1, below the 25th percentile of air pollutant concentrations (the reference group); Q2, the 25th to 50th percentile of air pollutant concentrations; Q3, the 50th to 75th percentile of air pollutant concentrations; Q4, above 75th percentile of air pollutant concentrations.
